# Anti-*blastocystosis* activity of antioxidant coated ZIF-8 combined with mesoporous silicas MCM-41 and KIT-6

**DOI:** 10.1038/s41598-022-10397-4

**Published:** 2022-04-17

**Authors:** B. Rabindran Jermy, Reem Y. Al-Jindan, Vijaya Ravinayagam, Ayman A. El-Badry

**Affiliations:** 1 Department of Nano-Medicine Research, Institute for Research and Medical Consultations (IRMC), Imam Abdulrahman Bin Faisal University, Dammam, Saudi Arabia; 2 Department of Microbiology, College of Medicine, Imam Abdulrahman Bin Faisal University, Dammam, Saudi Arabia; 3 Deanship of Scientific Research and Department of Nano-Medicine Research, Institute for Research and Medical Consultations, Imam Abdulrahman Bin Faisal University, P.O. Box 1982, Dammam, 31441 Saudi Arabia

**Keywords:** Microbiology, Medical research, Nanoscience and technology

## Abstract

The biocompatible hybrid Zeolitic imidazolate framework-8 (ZIF-8)/structured silica nanocomposite can be loaded with antioxidants such as curcumin and resveratrol to offer multiple advantages of drug functionalization and structural stability. *blastocystosis,* an enteric parasite, has various outcomes and its treatment includes drugs which have side effects and do not result in a full cure. We aimed to design novel biocompatible nanocomposites containing natural antioxidant, resveratrol or curcumin and ZIF-8/mesoporous silica. We also assessed their anti-*blastocystosis* activities as bioactive novel nanocomposites. The nano-silica (MCM-41 and KIT-6) was synthesized using a hydrothermal technique and made composite with ZIF-8 using an ultrasonic technique. The antioxidants, curcumin and resveratrol, were loaded over ZIF-8/MCM-41 and ZIF-8/KIT-6 using a rotary evaporator technique to form novel nanocomposites with bioactive properties. The formulated nanocomposites were characterized. To test their biological activity, suspension of cultured *blastocystosis* cysts (subtype 3) were exposed to increasing concentrations of nanocomposites and the minimal lethal concentration of each nanocomposite was calculated. The bioactive nanocomposites (ZIF-8/KIT-6, ZIF-8/KIT-6/Resveratrol and ZIF-8/MCM-41/Curcumin) were formulated. Anti-*blastocystosis* activity of the tested nanocomposites was both dose and time dependent. ZIF-8/KIT-6/Resveratol showed the maximum percentage of growth inhibition (~ 100%) at a concentration of 500 µg/ml after 5 h of exposure. More than 90% of *blastocystosis* cysts’ growth was significantly inhibited at all concentrations of ZIF-8/MCM-41/Curcumin, with different times of exposure, while it occurred only at the highest concentration of ZIF-8/KIT-6 (800 µg/ml). Using cheap, simple, reproducible and scalable techniques, we nano-formulated innovative bioactive nanocomposites, by incorporating the bioactive ZIF-8 (Zn^2+^ with imidazole), structured mesosilica and natural antioxidant compounds, curcumin or resveratrol, to generate multifunctional modalities. These eco-friendly, naturally based, safe, economical, biocompatible, and bioavailable nanocomposites are potential nanotherapeutics. The anti-*blastocystosis* results of these three nanocomposites indicate their potentially promising innovative and safe use as alternative *Blastocystosis* therapies.

## Introduction

Nanoparticles are one of the most significant discoveries of this past century; with direct therapeutic applications for human and animal health, they provide a promising improvement or replacement of current therapies for many medical disorders^1^. The most striking features of nanotherapeutics is their ability to accommodate several components into single nano structures, generating multifunctional modalities. Recently, the biomedical applications of Zeolitic Imidazolate Framework (ZIF-8), a type of metal organic materials with zeolitic morphology, has been widely studied^2^. The structure of ZIF-8 is composed of bioactive Zinc metal ion (Zn^2+^) with the pharmacologically active imidazole linker. The sodalite like structure is flexible, with a large surface area and structural stability. The presence of mesoporous structured silica, an organic/inorganic hybrid composite (ZIF-8/mesoporous silica), is reported to assist facile chemical functionalization, tunable porosity and in sustained drug release applications^3^. Rabindran et al.^4^ reported anticancer and antibacterial activity of Fe/SBA-16/ZIF-8 composite, mainly due to presence of ZIF-8 and cisplatin.

Structured mesoporous silica nanoplatforms like MCM-41 and SBA-15 (Santa Barbara Amorphous-15) are the most widely researched support in biomedical applications^5^. KIT-6 is a cubic shaped mesoporous silica with Ia3d symmetry^6^. The invention of hexagonal pore shaped MCM-41 and cubic shaped KIT-6 mesoporous carbon is used for catalysis, drug delivery and adsorption of biomolecules. The nanocarriers have attractive textural features such as high surface area with more than 1100 m^2^/g and pore size ranging between 1.5–10 nm with different types of pore orderings. Other structured silicates are SBA-1, SBA-16, and KIT-6^7,8^.

Resveratrol molecules are naturally present in grapes, blueberries, peanuts, and red currants. Resveratrol is safe for humans, with only minor side effects of gastrointestinal discomfort and diarrhea at high doses (2.5–5 g/day)^9^. Resveratrol molecules are a promising natural medicine, that may prevent cancer, protect against cardiovascular diseases, and are anti-inflammatory^9,10^, anti-mitotic^11^ and anti-microbial^12–14^.

Curcumin, a turmeric derivative, is obtained from the plant *Curcuma longa*. Curcumin is a polyphenol, which has potent antioxidant and a wide range of physiological, pharmacological and immuno-tissue protective effects, including anti-inflammatory, anti-amyloid, anti-neoplastic, anti-depressant, and anti-microbial as well as an immuno-modular and a regulator for metabolism. It has been traditionally used for treating various diseases^15–17^.

The ability of silica nanoparticles to conjugate natural polyphenols is of medicinal value and acts as a transferring agent for smuggling natural free radical scavengers across biological barriers^7,8^.

Parasitic diseases are a major public health threat in Saudi Arabia and worldwide. These diseases are responsible for considerable morbidity and mortality. *blastocystosis* is a unicellular parasitic protozoon, which has been detected globally, with up to 100% prevalence^18^. It inhabits the large intestine of humans and animals and is sometimes present in intestinal microbiomes with eubiosis^19^. Blastocystosis, human infection by *blastocystosis*, has various outcomes: it can be asymptomatic in infected patients, can produce diarrhea and other gastrointestinal symptoms, or have an opportunistic character and cause infection in immunocompromised patients. *blastocystosis* has been linked to symptoms of inflammatory bowel diseases, irritable bowel syndrome and acute urticaria^20–22^. Recently, *blastocystosis* was linked to gut dysbiosis and associated gut disorders as well as the induction of growth of colon and rectum cancer by apoptosis of cancer colon cells^23–25^.

Drugs currently used to treat *blastocystosis* are not fully effective. Among the many drugs used to treat *blastocystosis* infection, metronidazole is the most effective therapy, however, there remains controversy about responses of different categories of diarrheic patients, in addition to side effects and drug resistance^26^. Consequently, there is a vital need to develop effective alternative therapies to treat and control this disease.

The current study aimed to nano-formulate bioactive nanocomposites by incorporating several components, including structured nano-silica (MCM-41 or KIT-6), ZIF-8 and the natural antioxidants, curcumin and resveratrol, as safe innovative alternative therapeutics with multifunctional modality, and to assess the anti-*blastocystosis* activities of these nanotherapeutics.

## Materials and methodology

### Nanocomposites

Innovative bioactive naturally based polyphenols were nano-formulated, by accommodating ZIF-8, structured nano-mesosilica (MCM-41 or KIT-6) and the natural antioxidants, curcumin or resveratrol. ZIF-8 with textural properties of 1040 m^2^/g (Z1200, sigma Aldrich), was purchased and used as a composite with mesoporous silica. The hexagonal shaped nano-silica (MCM-41) and three-dimensional KIT-6 was synthesized using a hydrothermal technique. Cationic template cetyltrimethyl ammonium bromide (CTAB) and non-ionic template (P123) were used respectively to generate hexagonal and cubic pores for MCM-41 and KIT-6.

### Synthesis of MCM-41 and KIT-6

For synthesis of MCM-41; 10.6 g of sodium metasilicate (silica source) was taken and dissolved in 50 ml of distilled water and stirred vigorously for 1 h. After dissolution, the solution was added dropwise to 4.5 g of CTAB solution dissolved in 40 ml of distilled water. The mixture was further stirred for 2 h and then pH was adjusted to 10.5 using diluted sulphuric acid solution (4 N). The sol solution in milky form was hydrothermally treated at 140 °C for 12–24 h.

For synthesis of KIT-6; 4 g of P123 was dissolved in acidic HCl solution (2 M) and stirred for 1 h; then 4 g of *n*-butanol (co-solvent) was added along with 8.6 g of tetraethylorthosilicate (silica source) and stirred for 24 h. The mixture in polypropylene bottle was transferred to oven and hydrothermally aged at 100 °C for 24 h. The precipitate was filtered, washed several times with excess water and dried at 100 °C for 12 h. The two samples (MCM-41 and KIT-6) were finally calcined at 550 °C for 6 h.

The calcined form of MCM-41 and KIT-6 was composited with ZIF-8 using an ultrasonic technique forming ZIF-8/MCM-41 and ZIF-8/KIT-6 nanocomposite (ZIF-8/structured silica weight to 0.105 ratio). In order to nano-formulate with bioactive polyphenol components, a rotary evaporator was used. Curcumin or resveratrol (500 mg) were loaded over ZIF-8/MCM-41 and ZIF-8/KIT-6 (2000 mg) in methanolic solution (300 ml) and sonicated for 10 min; then the solvent was evaporated using rotary evaporator technique to form MCM-41/ZIF-8/antioxidant and KIT-6/ZIF-8/antioxidant nanocomposites. The formulated nanocomposites were characterized using phase (XRD), textural features (BET), chemical coordination environment of metal species (DRS-UV–visible), active component insertion (FTIR) and transmission electron microscopy (JEM2100F from JEOL).

### Drug release study

The release trend of antioxidants (resveratrol and curcumin) was investigated using four different nano-formulations MCM-41/ZIF-8/Curcumin, KIT-6/ZIF-8/Curcumin, MCM-41/ZIF-8/Resveratrol and KIT-6/ZIF-8/Resveratrol nanocomposites. Prior to the study, the dialysis membrane (12 kDa, Sigma Aldrich) was activated and then 15 mg of nanoformulation was dispersed in 25 ml of PBS solution (pH 5.6 and 7.4). The release of antioxidants was studied at 37 °C. At regular time intervals, 5 ml of solution was withdrawn, and release of antioxidants were measured using UV–visible spectroscopy. The withdrawn solution (5 ml) was replaced with equal volume of fresh PBS solution.

The release content was identified at the specific wavelength of curcumin and resveratrol (307 nm). Prior to analysis, calibration curve for curcumin and resveratrol was established. At first, an initial stock solution was prepared with concentration of 1000 µg/ml for curcumin and resveratrol. Various concentration of aliquots 5, 10, 15, 20, 25 and 30 µg/ml were prepared with makeup volume of 10 ml using release medium PBS solution (pH = 5.6 or 7.4) and calibration curve established against blank at maximum absorption wavelength λmax of 428 nm and 307 nm, respectively. Linear regression for curcumin and resveratrol were found to be y = 0.0041x + 0.0237 and y = 0.0579x + 0.0318, where y corresponds to absorbance and x to the concentration of antioxidant release (µg/ml). The correlation coefficient was of 0.9982 and 0.995 for maximum absorption for curcumin and resveratrol, respectively. The release study was repeated in triplicates.

### Biological activities of newly formulated nanocomposites

#### *blastocystosis* parasite

Suspension of cultured *blastocystosis* from symptomizing patients was used for evaluating biological activities of the formulated nanocomposites. *blastocystosis* were obtained from the El-Badry Lab and were ethically approved by the Deanship of Scientific Research and Postgraduate Studies of Imam Abdulrahman Bin Faisal University under the Institutional Review Board (IRB) number (IRB# 2021-01-009).

*blastocystosis* were sub-cultured using fresh Jones' medium enriched with 10% horse serum according to Jones^27^ to get rid of stool debris^28^. Cultures with vacuolar forms of *blastocystosis* greater than 10^6^/ml were used for evaluating biological activities of the formulated nanocomposites.

#### Molecular characterization of isolated *blastocystosis*

One ml of subculture was suspended in 7 ml PBS, vortexed, centrifuged for 1 min at 12,000×*g*, then the pellet was kept for DNA extraction. DNA was extracted from pelleted *blastocystosis* cysts, using commercial DNA extraction kit (DNA MiniPrep™ kit, Zymo Research Corp., USA) following the kit’s protocol. As per Stensvold’s^29^ recommendations, *blastocystosis* DNA was amplified and genotyped using two PCR reactions, targeting *blastocystosis* specific SSU rDNA^30^ and subtype-specific Sequence-Tagged-Site (STS)^31^ in order to detect the seven standardized subtypes (STs 1–7). PCR conditions and reactions were performed as mentioned by Yoshikawa et al.^30^ and Scicluna et al.^31^.

#### In vitro challenging of *blastocystosis* with nanocomposites

Suspension of cultured *blastocystosis* cysts, in logarithmic growth phase of 10^6^/ml, were inoculated in sets of culture tubes with Jones’ culture media. Haemocytometer counting chamber was used to count the number of *blastocystosis* cysts. The experiments contained 3 groups as follows:

- Group 1 (G1): parasite (negative, not treated) control group, containing only cultured cysts of *blastocystosis*.
- Group 2 (G2): drug (positive) control, containing cultured parasites exposed to 500 μg/ml metronidazole as a reference for anti- *blastocystosis* therapy.
- Group 3 (G3): nanocomposites tested group, containing cultured parasites exposed to the three nanocomposites. G3 was divided into subgroups to test three nanocomposites (G3A, G3B and G3C) at different concentrations (G3i-iv). Parasites were exposed to increasing concentrations of the three nanocomposites of 100 μg/ml (G3i) 200 μg/ml (G3ii), 500 μg/ml (G3iii) and 800 μg/ml (G3iv) (W/V). All tubes were incubated at 37 °C in humidified CO_2_ for 48 h.

All tested nanocomposites for each concentration were prepared by diluting stock suspension in appropriate amount of PBS. The volume of each of the experiment tubes was brought to 1 ml with *blastocystosis* 10^6^/ml concentration. All experiments were performed in triplicate^32–34^.

#### Assessment of anti-*blastocystosis* activities of the nanocomposites

The number of *blastocystosis* cysts from all cultured tubes included in the experiment were counted under the microscope in haemocytometer counting chamber, and cultured *blastocystosis* cysts were tested for their viability using Trypan blue solution (0.4%). All cultured tubes were examined for percentage of reduction in growth of *blastocystosis* cysts each hour for 5 h then after 24 h. The minimal lethal concentration (MLC) was determined to be the concentration at which no *blastocystosis* cysts were observed^32–34^.

### Statistical analysis

The obtained data was statistically analyzed using SPSS software and presented as mean and standard deviation (SD). All data shown represents the mean for each of the three independent experiments. Means were compared and variances were analyzed. *P* values of less than 0.05 indicate statistical significance.

### Ethical approval

The study protocol was reviewed by the Scientific Review Committee of Deanship of Scientific Research at the Imam Abdulrahman Bin Faisal University (Reference number: 2019-148-Med) and *blastocystosis* were obtained from cultured human stool where ethically approved by the Deanship of Scientific Research and Postgraduate Studies of Imam Abdulrahman Bin Faisal University under the Institutional Review Board (IRB) number (IRB# 2021-01-009).

## Results

**Figure 1:**
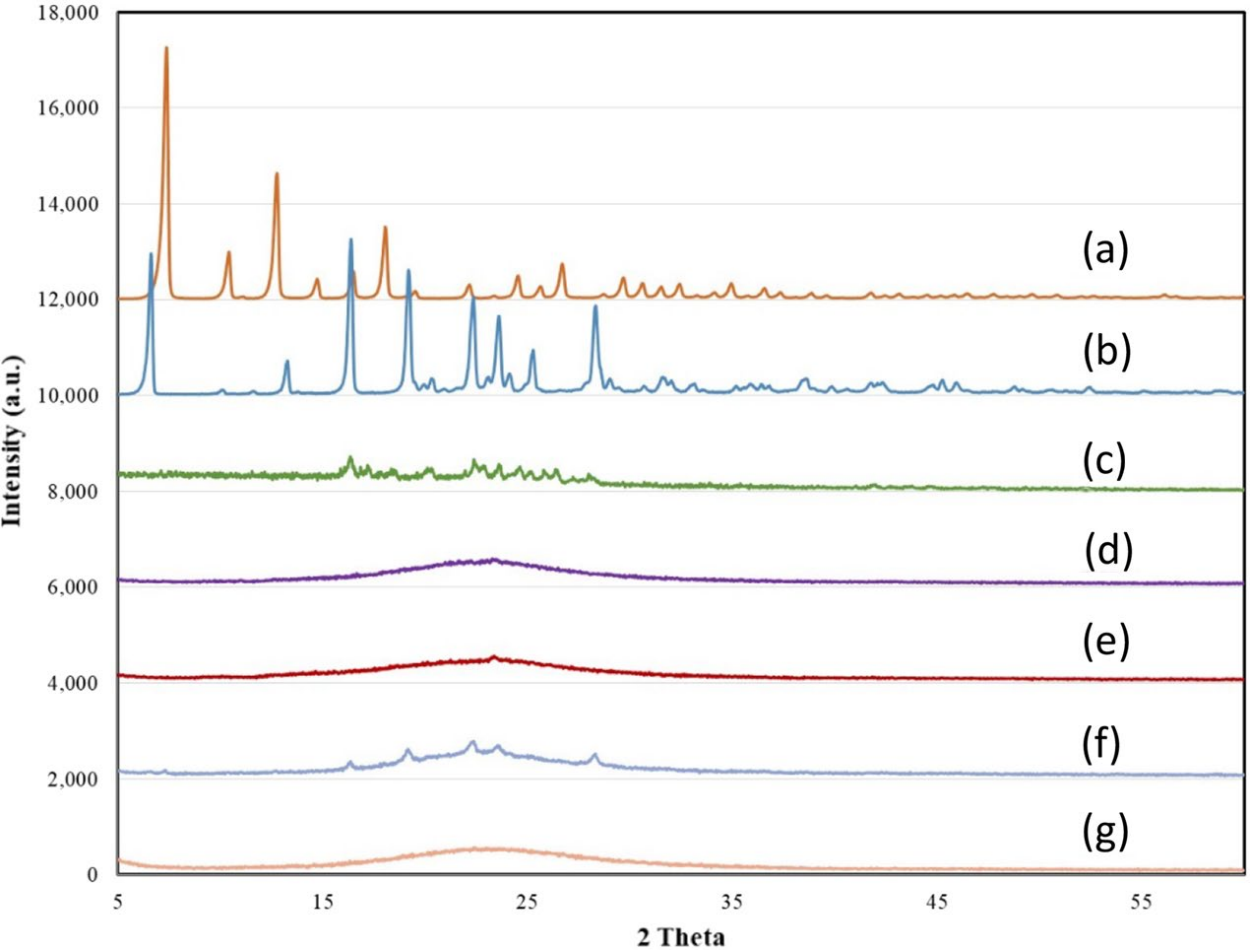
X-ray diffraction pattern of (a) ZIF-8, (b) Resveratrol, (c) Curcumin, (d) MCM-41/ZIF-8/Curcumin, (e) KIT-6/ZIF-8/Curcumin, (f) MCM-41/ZIF-8/Resveratrol and (g) KIT-6/ZIF-8/Resveratrol nanocomposites.

MCM-41/ZIF-8/Curcumin, KIT-6/ZIF-8/Curcumin, MCM-41/ZIF-8/Resveratrol and KIT-6/ZIF-8/Resveratrol were synthesized. The XRD analysis of ZIF-8, Resveratrol, Curcumin, MCM-41/ZIF-8/Curcumin, KIT-6/ZIF-8/Curcumin, MCM-41/ZIF-8/Resveratrol and KIT-6/ZIF-8/Resveratrol nanocomposites are shown in Fig. 1a–g.

**Figure 2:**
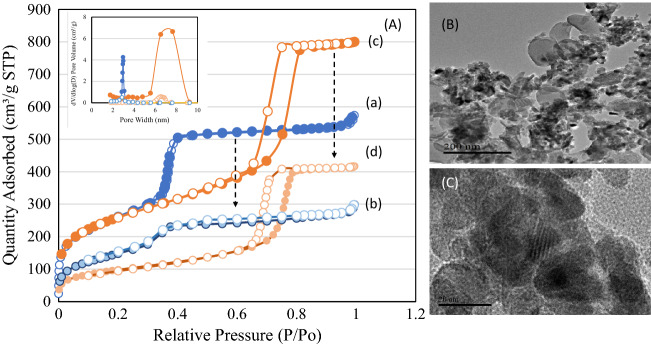
(**A**) Nitrogen adsorption isotherm pattern of (a) MCM-41, (b) MCM-41/ZIF-8, (c) KIT-6 and (d) KIT-6/ZIF-8. (**B**,**C**) Shows the transmission electron microscope images of KIT-6/ZIF-8 at magnification of 200 nm and 20 nm, respectively.

ZIF-8 exhibited typical crystalline peaks corresponding to sodalite structure (Fig. 1a). Resveratrol and Curcumin indicated a characteristics crystalline peak (Fig. 1b,c). Four samples of composites were analyzed to study the extent of crystallite transformation of ZIF-8 and bioactive components in contact with mesoporous silica. The characteristic peaks of ZIF-8 and curcumin were not observed in XRD spectra of MCM-41/ZIF-8/Curcumin and KIT-6/ZIF-8/Curcumin (Fig. 1d,e). These findings indicate the smaller crystallite size of ZIF-8 and the nanosize transformation of curcumin at the hexagonal and cubic pores of MCM-41 and KIT-6, respectively. In case of MCM-41/ZIF-8/Resveratrol, some peaks of resveratrol were seen, while in cubic shaped KIT-6 none were observed (Fig. 1f,g). These results show that some crystalline forms of resveratrol were still present inside the hexagonal pores of MCM-41. The transformation of ZIF-8 and antioxidants from crystalline to smaller crystallite size increased their bioavailability. The textural properties of MCM-41, KIT-6, MCM-41/ZIF-8 and KIT-6/ZIF-8 nanocomposites were measured using nitrogen adsorption technique (Fig. 2Aa–d). The surface area, pore volume and average pore size values are presented in Table 1. The adsorption–desorption isotherm pattern of MCM-41 and KIT-6 samples exhibited a typical type IV isotherm with capillary condensation due to large meso sized pores, at 0.2–0.4 and 0.6–0.8, respectively. The surface area of MCM-41 and KIT-6 was 942 m^2^/g and 897 m^2^/g, respectively; however, the formation of nanocomposite with ZIF-8 reduced the surface area of MCM-41/ZIF-8 and KIT-6/ZIF-8 to 594 m^2^/g and 336 m^2^/g. A similar trend in the pore volume and pore size distribution shows the effective interaction of structured silica with ZIF-8. The morphological analysis of KIT-6/ZIF-8 using TEM clearly shows the nanocomposite formation between KIT-6 and ZIF-8. The sphere shaped ZIF-8 was well distributed with large surface of KIT-6 (Fig. 2B,C).

**Table 1:** Textural and structural properties.

Sample	BET surface area (m^2^/g)	Pore volume (cm^3^/g)	Average pore size (nm)
MCM-41	942	0.87	3.7
KIT-6	897	1.24	5.5
MCM-41/ZIF-8	594	0.44	3.0
KIT-6/ZIF-8	336	0.64	7.6

**Figure 3:**
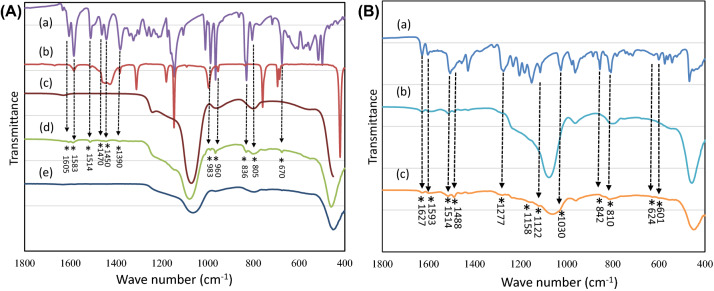
(**A**) FTIR spectra of (a) Resveratrol, (b) ZIF-8, (c) MCM-41, (d) MCM-41/ZIF-8/Resveratrol and (e) KIT-6/ZIF-8/Resveratrol nanocomposites. (**B**) FTIR spectra of (a) Curcumin, (b) MCM-41/ZIF-8/Curcumin and (c) KIT-6/ZIF-8/Curcumin nanocomposites.

Figure 3A shows the FTIR spectra of Resveratrol, ZIF-8, MCM-41, MCM-41/ZIF-8/Resveratrol and KIT-6/ZIF-8/Resveratrol nanocomposites. The spectra of resveratrol and ZIF-8 showed characteristic bands corresponding to carbon–carbon double bonds of aromatic compound at 1605, 1583 and 1514 cm^−1^ (Fig. 3Aa,b). Phenolic compound containing a carbonyl group showed a band at 1155 cm^−1^. The hydroxyl group from the phenolic compound showed a stretching at 1390 cm^−1^. The C-H group showed a band at 960 cm^−1^, indicating the trans resveratrol configuration. The C–H vibration band of arene conjugated to olefinic group can be seen at 805 cm^−1^ and 836 cm^−1^. In the various bands that are observed between 650–500 cm^−1^, a =C–H of olefinic group can be seen at 670 cm^−1^. Compared to functional bands of hexagonal pores of MCM-41 (Fig. 3Ac), various functional bands of resveratrol in reduced signals were observed, indicating the presence of an effective surface functionalization at the external pores (Fig. 3Ad); however, no such bands of resveratrol were observed in the cubic shaped pores of KIT-6 (Fig. 3Ae). This trend shows an effective pore filling of nanosized resveratrol in cubic pores of KIT-6. Figure 3B shows the FTIR spectra of curcumin, MCM-41/ZIF-8/Curcumin and KIT-6/ZIF-8/Curcumin nanocomposites (Fig. 3Ba–c). The functional groups of curcumin including > C=O and C=C were observed between 1627–1450 cm^−1^. In particular, a characteristic enolic OH of curcumin can be observed at 962 cm^−1^. The –C–O–C– chain vibrations (symmetric and asymmetric) can be observed between 1000–1450 cm^−1^ (Fig. 3Ba). In the case of both nanocomposites, the curcumin functional group showed a reduction in > C=O and C=C bands. A reduction in hydroxyl band of enol group also indicates an overall effective interaction of curcumin through enol and other functional groups (Fig. 3Bb,c). In the case of MCM-41 and KIT-6, the broadening of band at about 1023 cm^−1^ indicates the effective functionalization of curcumin in the hexagonal and cubic pores of nanocomposite. The presence of C–O–C band confirms the surface functionalization with the additional band appearance at 1030 cm^−1^.

**Figure 4:**
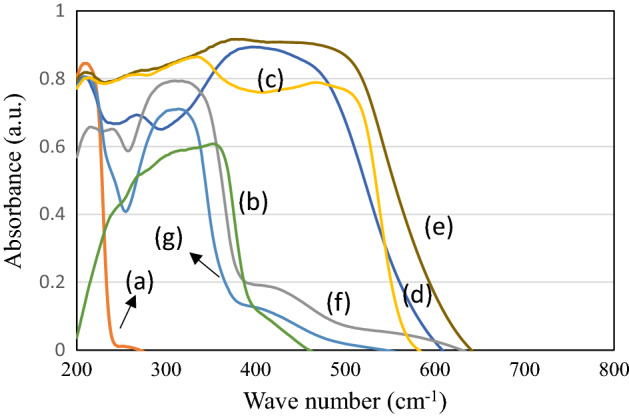
Diffuse reflectance spectra of (a) KIT-6/ZIF-8, (b) Resveratrol, (c) Curcumin, (d) MCM-41/ZIF-8/Curcumin, (e) KIT-6/ZIF-8/Curcumin, (f) MCM-41/ZIF-8/Resveratrol and (g) KIT-6/ZIF-8/Resveratrol nanocomposites.

Figure 4 shows the diffuse reflectance spectra of KIT-6/ZIF-8, Resveratrol, Curcumin, MCM-41/ZIF-8/Curcumin, KIT-6/ZIF-8/Curcumin, MCM-41/ZIF-8/Resveratrol and KIT-6/ZIF-8/Resveratrol nanocomposites. KIT-6/ZIF-8 shows a strong absorption at 212 nm, which can be ascribed to the presence of Zn^2+^ species in ZIF-8 (Fig. 4a). Resveratrol and curcumin revealed broad absorption between 200–600 nm (Fig. 4b,c). After loading of curcumin and resveratrol, the absorption maximum increases significantly over MCM-41/ZIF-8 and KIT-6/ZIF-8/nanocomposites. Such expansion behavior clearly indicates the composite formation over two supports (Fig. 4d–g).

### Drug delivery study

**Figure 5:**
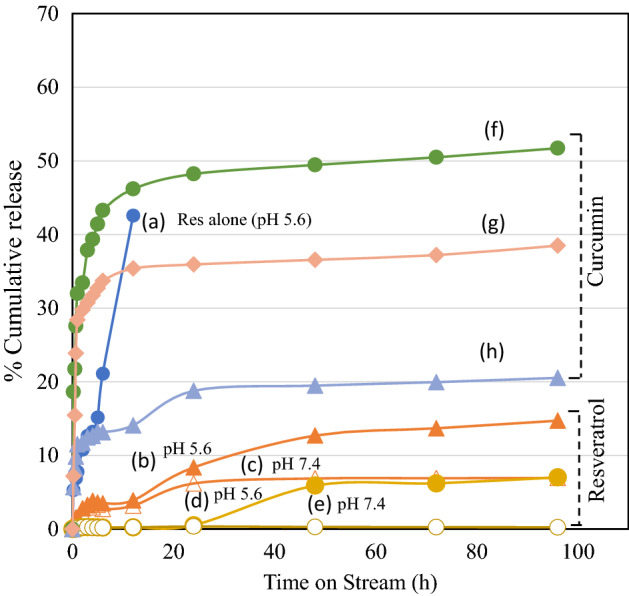
Release profile of antioxidants loaded nanocomposites at 37 °C for 96 h (a) Resveratrol (pH = 5.6), (b) MCM-41/ZIF-8/Resveratrol (pH = 5.6), (c) MCM-41/ZIF-8/Resveratrol (pH = 7.4), (d) KIT-6/ZIF-8/Resveratrol (pH = 5.6), (e) KIT-6/ZIF-8/Resveratrol (pH = 7.4), (f) KIT-6/Curcumin (pH = 5.6), (g) KIT-6/ZIF-8/Curcumin (pH = 5.6) and (h) MCM-41/ZIF-8/Curcumin (pH = 5.6).

The drug release ability of antioxidants on MCM-41/ZIF-8/Resveratrol and KIT-6/ZIF-8/Resveratrol nanocomposites was studied at intestinal parasite pH condition for human intestine, ranging in pH from 5.3 to 7.4 (Fig. 5). Resveratrol (pure form) was studied for comparative purpose. In the release study, as-such resveratrol in the absence of nanocarrier showed a burst release as expected and reached a maximum of 43% within 12 h. In the case of nanocomposite/resveratrol release profiles, MCM-41/ZIF-8 exhibited a slow release of about 15% for 96 h, while KIT-6/ZIF-8 showed even a lower release profile of about 7% for 96 h. At neutral pH condition, both nanocomposites showed a reduced release of resveratrol. Figure 5f shows the curcumin on KIT-6 support alone. A quick release of curcumin was observed reaching 37% within 3 h and reached 52% within 96 h. In case of nanocomposites, a wider difference was observed in curcumin release. KIT-6/ZIF-8 showed a higher percentage of cumulative release of curcumin (39%) than MCM-41/ZIF-8 (21%) at pH 5.6.

*blastocystosis*-DNA was amplified using the primers targeting *blastocystosis* specific SSU rDNA sequence of *blastocystosis* STs. *blastocystosis* DNA was amplified and genotyped as subtype-3 using the primers targeting *blastocystosis* subtype-specific STS.

**Figure 6:**
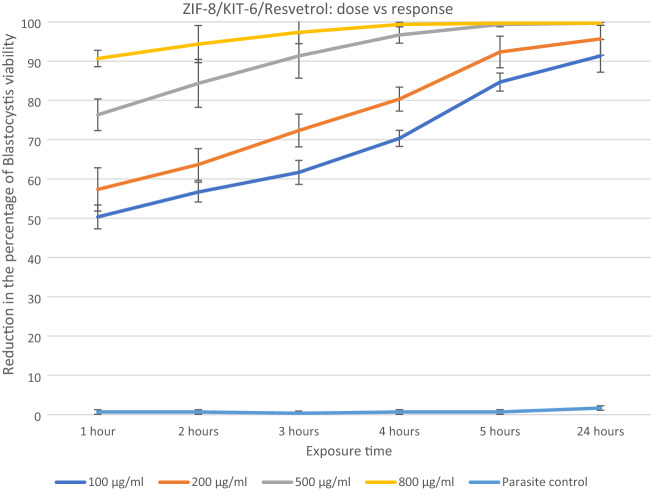
Reduction in the percentage of *blastocystosis* cysts viability after exposure to increasing concentrations of ZIF-8/Kit-6/Resveratrol nanocomposite.

**Figure 7:**
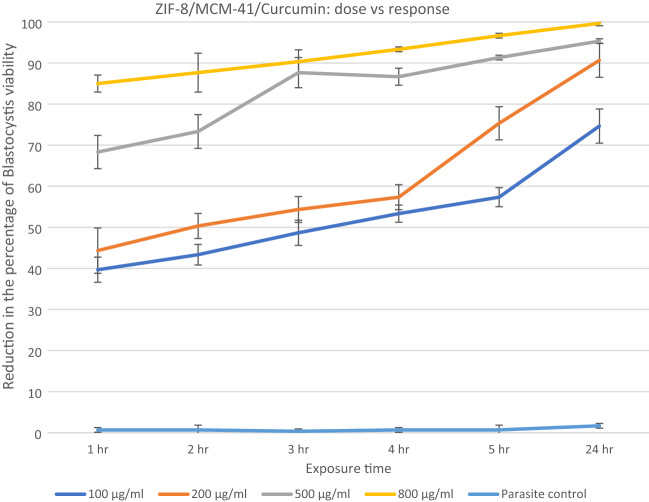
Reduction in the percentage of *blastocystosis* cysts viability after exposure to increasing concentrations of ZIF-8/MCM-41/Curcumin nanocomposite.

**Figure 8:**
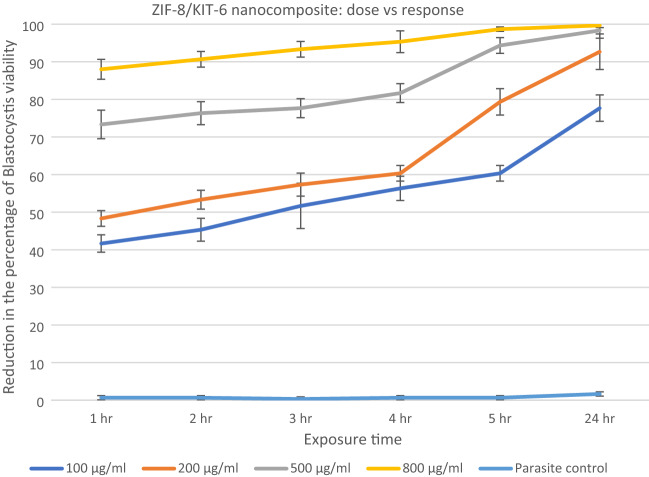
Reduction in the percentage of *blastocystosis* cysts viability after exposure to increasing concentrations of ZIF-8/KIT-6 nanocomposite.

The anti-*blastocystosis* cysts activities of the three nanocomposites, ZIF-8/MCM-41/Curcumin, ZIF-8/KIT-6/Resveratrol and ZIF-8/KIT-6 were determined by calculating the viability percent of *blastocystosis* cysts exposed to different increasing concentrations of all the nanocomposites each hour for 5 h then after 24 h (100, 200, 500 and 800 µg/ml). The parasite control group showed no effect on viability of *blastocystosis* cysts, while there was a reduction in number of cysts exposed to the nanocomposite; the maximum effect was observed with ZIF-8/MCM-41/Resveratrol (Figs. 6, 7, 8 and Table 2).

**Table 2:** Reduction in the percentage of viability of *blastocystosis* cysts in parasite control group (negative control), after exposure to metronidazole (positive control), and after exposure to the three nanocomposites.

	Time of exposure (h)					
	1 h	2 h	3 h	4 h	5 h	24 h
Parasite control	0.67 ± 0.58	0.67 ± 1.15	0.33 ± 0.58	0.67 ± 0.58	0.67 ± 1.15	1.67 ± 0.58
Metronidazole control	81.67 ± 2.52	87.67 ± 1.53	91.33 ± 1.53	96.33 ± 1.15	98.67 ± 0.58	99.33 ± 0.58
**ZIF-8/KIT-6/Resvetrol**						
100 µg/ml						
Mean ± Sd	50.33 ± 3.06	56.67 ± 2.52	61.67 ± 3.06	70.33 ± 2.08	84.67 ± 2.31	91.33 ± 4.16
* P* value	0.0004*	0.0002*	0.0005*	0.0004*	0.0003*	0.0003*
200 µg/ml						
Mean ± Sd	57.33 ± 5.51	63.67 ± 4.04	72.33 ± 4.16	80.33 ± 3.06	92.33 ± 4.04	95.67 ± 4.16
* P* value	0.001*	0.001*	0.0007*	0.0003*	0.0003*	0.004*
500 µg/ml						
Mean ± Sd	76.33 ± 4.04	84.33 ± 6.11	91.33 ± 5.68	96.67 ± 2.08	99.33 ± 0.58	99.67 ± 0.58
* P* value	0.0004*	0.0006*	0.0008*	0.0001*	0.04*	0.05*
800 µg/ml						
Mean ± Sd	90.67 ± 2.08	94.33 ± 4.73	97.33 ± 2.89	99.33 ± 0.58	99.67 ± 0.58	99.67 ± 0.58
* P* value	0.008*	0.0003*	0.0001*	0.21	0.42	0.12
**ZIF-8/MCM-41/Curcumin**						
100 µg/ml						
Mean ± Sd	39.67 ± 2.3	43.34 ± 3.05	48.67 ± 6.03	53.34 ± 3.21	57.34 ± 2.08	74.67 ± 3.51
* P* value	0.0004*	0.0002*	0.0005*	0.0004*	0.0003*	0.0003*
200 µg/ml						
Mean ± Sd	44.34 ± 2.08	50.34 ± 2.52	54.34 ± 3.06	75.34 ± 2.08	84.34 ± 3.51	90.67 ± 4.73
* P* value	0.001*	0.001*	0.0007*	0.0003*	0.0003*	0.001*
500 µg/ml						
Mean ± Sd	68.34 ± 3.79	73.34 ± 3.06	78.34 ± 2.52	86.67 ± 2.52	91.33 ± 2.08	95.34 ± 2.08
* P* value	0.0004*	0.0006*	0.0008*	0.0001*	0.04	0.0001*
800 µg/ml						
Mean ± Sd	85.00 ± 2.65	87.67 ± 2.08	90.34 ± 2.08	93.33 ± 2.89	96.67 ± 0.58	99.67 ± 0.58
* P* value	0.008*	0.0003*	0.0001*	0.21	0.42	0.0001*
**ZIF-8/KIT-6**						
100 µg/ml						
Mean ± Sd	36.33 ± 0.58	40.33 ± 1.15	44.67 ± 1.15	49.33 ± 0.58	56.67 ± 0.58	63.33 ± 1.15
* P* value	0.0003*	0.0006*	0.0008*	0.001*	0.005*	0.035
200 µg/ml						
Mean ± Sd	41.67 ± 1.53	45.67 ± 1.53	51.33 ± 0.58	56.33 ± 0.58	62.67 ± 0.58	75.67 ± 1.53
* P* value	0.003*	0.002*	0.003*	0.0045	0.034	0.135
500 µg/ml						
Mean ± Sd	49.33 ± 0.58	54.67 ± 1.15	58.67 ± 1.53	64.67 ± 1.53	78.33 ± 2.08	85.33 ± 0.58
* P* value	0.005*	0.021*	0.053*	0.047*	0.033*	0.211
800 µg/ml						
Mean ± Sd	72.67 ± 1.15	76.33 ± 3.06	77.67 ± 2.52	81.67 ± 2.52	86.67 ± 2.08	91.33 ± 0.58
* P* value	0.008*	0.100	0.021	0.072	0.15	0.40

Data are presented as the mean ± standard deviation (SD), *P < 0.05 is statistically significant, compared to the parasite control.

ZIF-8/KIT-6/Resveratrol showed the maximum percentage of *blastocystosis* cysts destruction (~ 100%) at concentration of 500 µg/ml after 5 h of exposure, as found using standard drug (metronidazole) with statistical significance.

All of the ZIF-8/KIT-6/Resveratrol nanocomponent tested concentrations and three concentrations (200, 500 and 800 µg/ml) of the ZIF-8/MCM-41/Curcumin as well as highest concentration (800 µg/ml) of ZIF-8/KIT-6 killed more than 90% of the viable *blastocystosis* cysts in one day compared to parasite control, untreated *blastocystosis* cysts. The percentage of viability of *blastocystosis* cysts steadily declined over the experiment time; it was dose-dependent in all tested nanocomposites, with strongest effect using resveratrol (Figs. 6, 7, 8 and Table 2). There was a statistical significance in low doses of the nanocomposites (Table 2).

**Figure 9:**
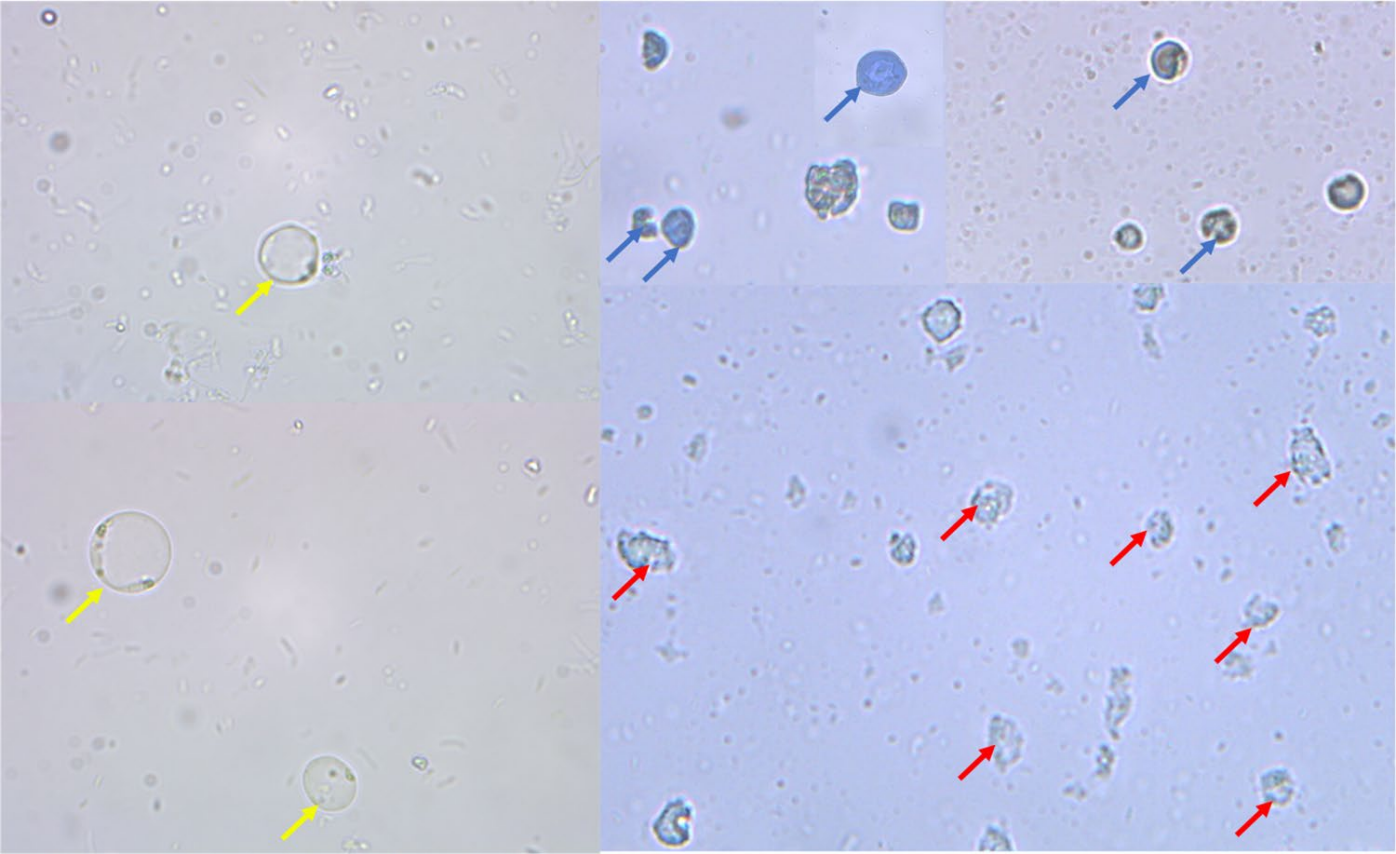
Trypan blue dye exclusion stained *blastocystosis* cysts, 400 × magnification. Live *blastocystosis* cysts (yellow arrows) (control group). Dead *blastocystosis* cysts (blue arrows). Disintegrated *blastocystosis* cysts (red arrows).

Trypan blue is a vital stain which does not stain a live *blastocystosis* cysts (Fig. 9), while it stains dead *blastocystosis* cysts blue (Fig. 9). The dead *blastocystosis* cysts were further confirmed by microscopic detection of cell wall disruption and destruction of internal structures.

## Discussion

### Nanocomponents are at the forefront of the promising and expanding field of nanotechnology

Due to their highly crystalline nature, the antioxidants resveratrol and curcumin, are insoluble in water, and consequently of reduced bioavailability and poorly absorbed when taken orally^1,35^. The advantage of using a nanocarrier (nano-silica) with nano-size pores is its ability to transform the bioactive components from an insoluble state (crystalline state) into a nanoform soluble state (amorphous state), thus increasing their bioavailability^1,8^.

In the current study, resveratrol and curcumin was nano-formulated and loaded to ZIF-8/KIT-6 and ZIF-8/MCM-41, a metal organic framework (MOF) built with bioactive and biocompatible components ZIF-8 and silica. This crystallite size transformation of ZIF-8 and the antioxidants resveratrol and curcumin amorphous form, enhanced their biocompatibility and bioavailability. Curcumin and resveratrol built with the two bioactive components (ZIF-8/MCM-41 and ZIF-8/KIT-6), work synergistically as antiparasitic nanotherapeutics.

MOFs are shown to exhibit pH sensitive drug releases. The degradation of MOF at acidic, neutral, and basic conditions tends to influence drug release characteristics. In the current study, the nanocarrier showed high drug loading efficiency and pH sensitive release capability due to inherent anchoring nature of metal–oxygen clusters. The high release at acidic pH of 5.5 could be attributed to the protonation of drug leading to a higher release than at the neutral pH of 7.4^36,37^. The ZIF-8 nanocarrier with pH sensitivity has been reported to be beneficial against parasite protozoan *Trypanosoma* infection. At acidic pH of 4.5, a quick release of benznidazole was observed, while the trend changes to a sustained and longer release at pH 7.4^38^. In our case, the hydroxyl functionalization of curcumin and resveratrol with nanocomposites may be critical in protonation and might assist such release in acidic pH condition. Both MCM-41/ZIF-8 and KIT-6/ZIF-8 nanocomposites showed a reduction in surface area and hierarchical pore formations compared to MCM-41 and KIT-6. In parallel, an intermediate pH sensitive release with steady modulation of antioxidants occurs in KIT-6/ZIF-8 more than with KIT-6 and resveratrol.

To date, there is no full cure for Blastocystosis, an emerging parasitic disease^26,39^. Small doses of metronidazole (as of 10 μg/ml) have been used as in-vitro anti-blastocystosis therapy^40,41^; however, several studies stated the need for higher doses up to 1 mg/ml^40–43^. In the current study, we used a higher dose of 500 μg/ml of metronidazole.

Resveratrol has shown promising anti-protozoal activity, but its anti-*blastocystosis* activity has yet to be evaluated. In the current study, we assessed for the first time the in vitro effects of resveratrol and ZIF-8/KIT-6 nanocomponents on the viability of *blastocystosis* cysts. The current study showed that there was dose-dependent, and time depended anti-*blastocystosis* cysts activity for the two assessed nanocomposites at low concentrations. The MLC of resveratrol nanocomponents was 100 μg/ml, which is a much smaller dose than higher doses previously tested^44^. Juan et al.^44^ reported that the prolonged eating (for 28 days) of large doses of resveratrol (1.4 g), 1000 times the amount ingested daily by a human of 70 kg, caused no harm to rats when compared to rat control group.

The current study tested the anti-*blastocystosis* activity of nanocomponents (curcumin, resveratrol, KIT-6 and ZIF-8/MCM-41), which were used in higher doses in previous studies and proven to be non-cytotoxic for human cells^9,44–46^.

We nano-formulated innovative nanocomposites, using cheap, simple, reproducible, and scalable techniques. They are biocompatible, bioavailable, stabile in acidic environments, eco-friendly, safe and economical naturally based compounds that can be used as alternative anti-microbial therapeutics.

Many studies have attributed the anti-protozoal activities of resveratrol and curcumin to reduced oxygen consumption by protozoa^9,47–49^, which may explain its mechanism of action as anti-*blastocystosis*. There are many advantages to the nano-scaling of resveratrol and curcumin. It enhances their therapeutic activities by inducing cellular stress responses at lower doses. This low dose adaptive response is due to its nano-size with large surface area to volume ratio^13^. Giving the drug in lower doses reduces toxicity^13^. Remarkably, with prolonged exposure, all nanocomposites killed *blastocystosis* cysts, this may be because nanocomposites accumulate in host cells and their action is sustained for a longer time.

In the current study, Trypan blue vital stain enabled easy differentiation between viable and non-viable cysts, similarly several previous studies reported the reliability of Trypan blue stain in counting the viability of *blastocystosis*^32,34,40,50^. The *blastocystosis* clinical isolates from symptomatic patients used for our experiment were *blastocystosis* subtype 3. This tested strain was susceptible to the anti-*blastocystosis* effects of the nanocomposites used, particularly resveratrol.

## Conclusions

Using cheap, simple, eco-friendly, reproducible, and scalable techniques, we nano-formulated innovative nanocomposites, incorporating three distinct components: bioactive ZIF-8, structured mesosilica, and curcumin or resveratrol (safe and natural medicinal antioxidants).

These nanocomposites are biocompatible, bioavailable, economical and are stabile in acidic environments, making them promising alternative anti-microbial therapeutics.

The obtained findings provide first time evidence that ZIF-8/KIT-6/Resveratrol in addition to curcumin has anti-*blastocystosis* activity and destroys *blastocystosis* cysts in vitro with small MLC. The encouraging anti-*blastocystosis* results for these three nanotherapeutics suggest that they are safe and innovative alternative blastocystosis therapies. Further research is required to test their in vivo effect and to assess the anti-microbial activities of the formulated nanocomposites against other microbes.
